# Indirect mitral annuloplasty in patients with reduced or preserved ejection fraction: A real‐world, single‐centre experience

**DOI:** 10.1002/ehf2.70016

**Published:** 2025-11-19

**Authors:** Holger Priebe‐Brämer, Firas Jaly, Nithusa Rahunathan, Mark Luedde, Alexander Albert, Klaus K. Witte, Hans‐Joerg Hippe

**Affiliations:** ^1^ Department of Cardiology Marien Hospital Witten Witten Germany; ^2^ Hull Teaching Hospitals NHS Trust Kingston‐upon‐Hull UK; ^3^ Medical Clinic 1, Cardiology and Angiology Justus‐Liebig University Giessen Germany; ^4^ Department of Cardiac Surgery Klinikum Dortmund Dortmund Germany; ^5^ Witten/Herdecke University Witten Germany; ^6^ Leeds Institute of Cardiovascular and Metabolic Medicine University of Leeds Leeds UK; ^7^ Christian‐Albrechts University of Kiel Kiel Germany

**Keywords:** heart failure, mitral annuloplasty, natriuretic peptides, secondary mitral regurgitation

## Abstract

**Aims:**

We aim to evaluate the real‐world effectiveness and safety of the Carillon Mitral Contour System (CMCS) for indirect annuloplasty in patients with heart failure (HF) and secondary mitral regurgitation (SMR).

**Methods and results:**

This was a single‐centre retrospective study of 204 consecutive patients (age 83 ± 6 years, 68% female, 72% HFrEF) with NYHA class II‐IV HF and moderate and moderate‐to‐severe (grade 2 + or 3+) SMR planned for CMCS implantation between 2021 and 2024. Echocardiographic variables, B‐type natriuretic peptide (BNP) and NYHA functional class were routinely assessed prior to the procedure and, in those with a successful implantation, after 3 months. The device was successfully implanted in 201 (98.5%) patients; the remaining 3 had unsuitable coronary sinus anatomy. Procedural complications occurred in 10.4% and included circumflex artery compression requiring percutaneous coronary intervention (9.5%); coronary sinus injury (1.5%); procedural mortality (0%). At 3 months, NYHA class improved by ≥1 category in 94% of patients and 98% achieved NYHA class I or II. MR decreased by ≥1 grade in 91% of patients, with all patients classified as grade 1+ or 2+ post‐procedure. *Vena contracta* width decreased (6.43 ± 0.84 to 3.85 ± 1.19 mm; *P* < 0.001), and there was a reduction of LA area from 44.4 ± 6.0 to 35.4 ± 5.1 cm^2^ (*P* < 0.001). Diastolic filling was improved (change in E/E′ ratio from 20.1 ± 5.5 to 14.0 ± 3.8; *P* < 0.001), systolic pulmonary artery pressure was lower in follow‐up (44 ± 11 to 35 ± 9 mm; *P* < 0.001), and there was also a clinically relevant reduction in median BNP levels from 2849 to 1390 pg/mL (*P* < 0.001). These changes were not different between HFrEF and HFpEF.

**Conclusions:**

In this single‐centre observational cohort study, CMCS implantation success was high, and associated with low complication rates, improvements in symptoms and echocardiographic variables, and clinically relevant reductions in BNP levels.

## Introduction

Secondary mitral regurgitation (SMR) is a common finding in people with heart failure with reduced (HFrEF) or preserved (HFpEF) ejection fraction.^1^ SMR is commonly the expression of either ventricular or atrial remodelling with consequent annular dilatation, papillary muscle displacement and failure of adaptation of otherwise normal mitral leaflets.^2^ This physiological cascade accelerates HF progression through reduced forward cardiac output, increased left atrial (LA) pressure, and pulmonary hypertension. Even mild SMR is associated with worse symptoms, worse cardiac function and augurs a worse prognosis in patients with both HF phenotypes, including those receiving optimal guideline‐directed medical therapy (GDMT).^3^, ^4^, ^5^

In the face of limited prognostic benefit of surgical options for SMR,^6^, ^7^, ^8^, ^9^ and the growing number of multi‐morbid patients with SMR in the presence of either HFpEF or HFrEF, percutaneous therapeutic interventions for SMR have evolved substantially. Transcatheter edge‐to‐edge repair (TEER) is a minimally invasive technique with generally favourable but inconsistent results in open‐label, randomized trials, where patients presenting with severe left ventricular (LV) dysfunction or greater LV dilatation derived less benefit.^10^, ^11^, ^12^ While TEER effectively reduces MR by improving leaflet coaptation, it does not consistently address other important mechanisms of SMR such as annular dilation and does not appear to promote beneficial remodeling, even in patients with LV dilatation.^13^ This therapeutic gap has stimulated interest in percutaneous annuloplasty techniques that directly address annular dilation in SMR.

The Carillon Mitral Contour System (CMCS; Cardiac Dimensions, Inc., Kirkland, WA, United States) is a percutaneous indirect annuloplasty device that leverages the anatomical proximity between the coronary sinus and posterior mitral annulus. By shortening the superior and posterior coronary sinus, the device reduces the dimensions of the mitral annulus, thereby improving SMR. In controlled trials in HFrEF patients, the CMCS leads to improvements in LV volume, MR severity and functional status,^14^, ^15^, ^16^, ^17^, ^18^, ^19^ including patients less likely to benefit from TEER.^20^ Despite this, the real‐world clinical performance of Carillon therapy warrants additional study, particularly in patients with HFpEF who were excluded from these trials and in whom the pathophysiology and treatment response may differ from those with HFrEF. Therefore, this study aimed to evaluate the echocardiographic, haemodynamic and clinical outcomes following CMCS implantation in a large, real‐world patient cohort treated at a single centre, with key results stratified by HF phenotype.

## Methods

This retrospective, observational study evaluated the outcomes of unselected, consecutive patients with at least moderate SMR who underwent CMCS implantation at a single centre over a 4‐year period from 2021.

### Patients

Patients were those treated in routine clinical practice, with selection criteria reflecting published evidence and national guideline‐based echocardiographic features, combined with patient clinical status assessed and discussed by the Heart Team to confirm eligibility. Patients with symptomatic HF (New York Heart Association (NYHA) functional class II–IV) and with echocardiographically confirmed grade 2+ or 3+ SMR despite GDMT were included. Exclusion criteria were limited to device‐specific anatomical contraindications including pre‐existing cardiac resynchronisation therapy and previous mitral valve surgery. Notably, no restrictions were imposed on LV dimensions or ejection fraction (LVEF), thereby allowing outcome evaluation in HFrEF and HFpEF patients.

### Procedure

The CMCS utilizes a catheter‐based approach to perform indirect annuloplasty through mechanical cinching of the mitral valve annulus. The implantation procedure has been described in detail elsewhere.^14^ Briefly, the device is delivered through the right internal jugular vein into the coronary sinus; one anchor is placed into the distal coronary sinus, tension is applied and a further anchor is deployed near the coronary sinus ostium to maintain the plication. Assessment of coronary arterial flow is performed before final device release to ensure the absence of compression by the anchors or the ribbon. Implantation can occur under general anaesthesia or conscious sedation, with the approach individualized based on patient characteristics and physician preference.

### Outcomes

Procedural outcomes included procedure time, fluoroscopy time, contrast volume and radiation dose‐area product. Safety outcomes included immediate, in‐hospital and 30‐day procedure‐related complications, mortality and hospital length of stay. Patient‐orientated outcomes focused on symptoms assessed by the NYHA status. Mechanistic outcomes included echocardiographic variables and BNP, each assessed at baseline and 3 months. Echocardiographic variables included left ventricular end diastolic diameter (LVEDD), left atrial dimensions, *vena contracta* width, MR grade according to the American Society of Echocardiography guidelines,^21^ diastolic function (E/E′ ratio, E‐wave velocity) and systolic pulmonary artery pressure.

### Statistical analysis

Baseline patient characteristics and echocardiographic data were analysed using descriptive statistics and divided by HF phenotype (HFpEF LVEF ≥ 50%; HFrEF LVEF < 50%). Continuous variables with a normal distribution are presented as mean with standard deviation (SD) while non‐normally distributed data are reported as median with interquartile range (IQR). Categorical variables are presented as counts and percentages. Comparisons between HF phenotypes were analysed using the independent samples *t*‐test, Wilcoxon–Mann–Whitney test or Fisher's exact test.

We assessed complication rates using the learning curve cumulative sum (LC‐CUSUM) method, which tracks the cumulative difference between complication rates in consecutive cases and the overall rate to identify learning, plateau and proficiency phases.

The mean change and 95% confidence interval (95% CI) in results from baseline to 3‐month follow‐up were analysed using paired samples t‐tests for normally distributed data (LVEDD, left atrial dimensions, E/E′ ratio, E‐wave velocity, systolic pulmonary artery pressure, *vena contracta* width). The Wilcoxon signed‐rank test was used to analyse non‐parametric and ordinal data (NYHA class, MR grade, BNP), with the median change and 95% CI in BNP calculated using the Hodges‐Lehmann estimator. Phenotype subgroups were compared using analysis of covariance or quantile regression with adjustment for the baseline value. Firth logistic regression was used to identify variables independently associated with improvement in NYHA class (≥1 class) and MR grade (≥1 grade) over 3 months. All tests were two‐sided with a threshold of *P* < 0.05 for statistical significance. Statistical analyses were performed using Stata v19.5 (StataCorp, College Station, TX, United States).

The institutional ethics committee of the University of Witten/Herdecke approved data collection for the present analysis study, and all participants prospectively provided written, informed consent for the implant procedure and follow‐up assessments. All study procedures adhered to principles outlined in the Declaration of Helsinki and followed institutional guidelines for human research.

## Results

### Patient characteristics

Between January 2021 and July 2024, 204 consecutive patients, deemed appropriate for CMCS implantation by the multi‐disciplinary Heart Team, based upon the available evidence, underwent attempted CMCS implantation. Of these, 201 (98.5%) successfully received the device. Three patients were not implanted with the Carillon device due to coronary sinus anatomy that prevented successful device deployment.

*Table* *1* shows the demographics, LV function and co‐morbidities of the 201 implanted patients. Mean age was 83 ± 6 years and 68% were female. Mitral regurgitation was classed as grade 2 + (55%) or 3 + (45%) and most (95%) had NYHA class III or IV symptoms. Mean LVEF was 44 ± 8% and 72% had HFrEF. Atrial fibrillation (AF) was a common co‐morbidity (80%) and coronary artery disease was present in 69%. Nearly two‐thirds (61%) had evidence of important diastolic dysfunction. Compared to HFpEF, the HFrEF subgroup had a lower proportion of females (*P* = 0.04), a higher prevalence of coronary artery disease (*P* = 0.004), higher EuroScore II score (*P* = 0.01), and higher BNP levels (*P* < 0.001).

**Table 1:** Baseline patient characteristics

Variable	Total (*n* = 201)	HFrEF (*n* = 144)	HFpEF (*n* = 57)	*P*‐value
Age (years)	83 ± 6	83 ± 6	84 ± 5	0.41
Female sex (%) [*n*]	68.2 (137/201)	63.9 (92/144)	78.9 (45/57)	0.04
Atrial fibrillation (%) [*n*]	79.6 (160/201)	82.6 (119/144)	71.9 (41/57)	0.12
Coronary artery disease (%) [*n*]	68.7 (138/201)	75.0 (108/144)	52.6 (30/57)	0.004
Previous cardiac surgery (%) [*n*]	14.4 (29/201)	16.7 (24/144)	8.8 (5/57)	0.19
EuroScore II	8.4 ± 5.0	9.0 ± 5.3	7.0 ± 3.7	0.01
BNP (pg/mL)^a^	2861 (1308, 5893)	3339 (1814, 6929)	1581 (670, 3255)	<0.001
NYHA class (%) [*n*]				0.59
II	4.5 (9/201)	5.6 (8/144)	1.8 (1/57)	
III	84.6 (170/201)	84.0 (121/144)	86.0 (49/57)	
IV	10.9 (22/201)	10.4 (15/144)	12.3 (7/57)	
Echocardiographic variables				
LV ejection fraction (%)^b^	44 ± 8	40 ± 7	52 ± 4	<0.001
LVEDD (mm)	52 ± 7	53 ± 7	50 ± 6	0.002
LA area (cm^2^)	44 ± 6	45 ± 6	43 ± 5	0.006
PAP systolic (mmHg)	44 ± 11	44 ± 11	42 ± 11	0.10
MR grade (%) [*n*]				0.28
2+	55.2 (111/201)	52.8 (76/144)	61.4 (35/57)	
3+	44.8 (90/201)	47.2 (68/144)	38.6 (22/57)	
Vena contracta (mm)	6.4 ± 0.8	6.5 ± 0.9	6.3 ± 0.8	0.22
Diastolic dysfunction (%) [*n*]				0.62
Grade I	6.0 (12/201)	6.3 (9/144)	5.3 (3/57)	
Grade II	33.3 (67/201)	31.3 (45/144)	38.6 (22/57)	
Grade III	60.7 (122/201)	62.5 (90/144)	56.1 (32/57)	
Tricuspid regurgitation (≥2+)	88.1 (177/201)	90.3 (130/144)	82.5 (47/57)	0.15

Values are mean ± standard deviation, or percentage (*n*/*N*) unless noted otherwise. Diastolic dysfunction was defined as Grade I: E/A < 0.8, Grade II E/A 0.8–1.5, E/E′ > 15, s > D and Grade III E/A 1.8–3.0, E/E′ > 15 and s > D.

BNP, B‐type natriuretic peptide; HFpEF, heart failure with preserved ejection fraction; HFrEF, heart failure with reduced ejection fraction; LV, left ventricular; MR, mitral regurgitation; NYHA, New York Heart Association.

^a^ Values are median (interquartile range).

^b^ LV ejection fraction <50% indicates HFrEF; ≥50% indicates HFpEF.

### Procedural data and safety endpoints

All implantation procedures took place under conscious sedation. The median procedure time was 63 min (IQR 51–113), fluoroscopy time was 16 min (IQR 12–24), and contrast volume was 95 mL (IQR 64–131).

Immediate procedural complications occurred in 10.4% of cases and included circumflex artery compression to >50% (9.5%), all of which were successfully treated with percutaneous coronary intervention, and coronary sinus injury resulting in pericardial effusion (1.5%), none of which had long‐term consequences (*Table* *S1*). There were no strokes and no clinically significant bleeding complications. No learning curve for complications was detected (*Figure* *S1*), with 30‐day complication rates ranging between 5.3% and 15.4% over the course of the 4 years. The median hospital stay was 3 days (IQR 3–4 days), with no operative mortality. There were no important differences in procedural variables or complication rates between HF phenotypes (*Table* *S1*).

### Follow‐up data

**Figure 1:**
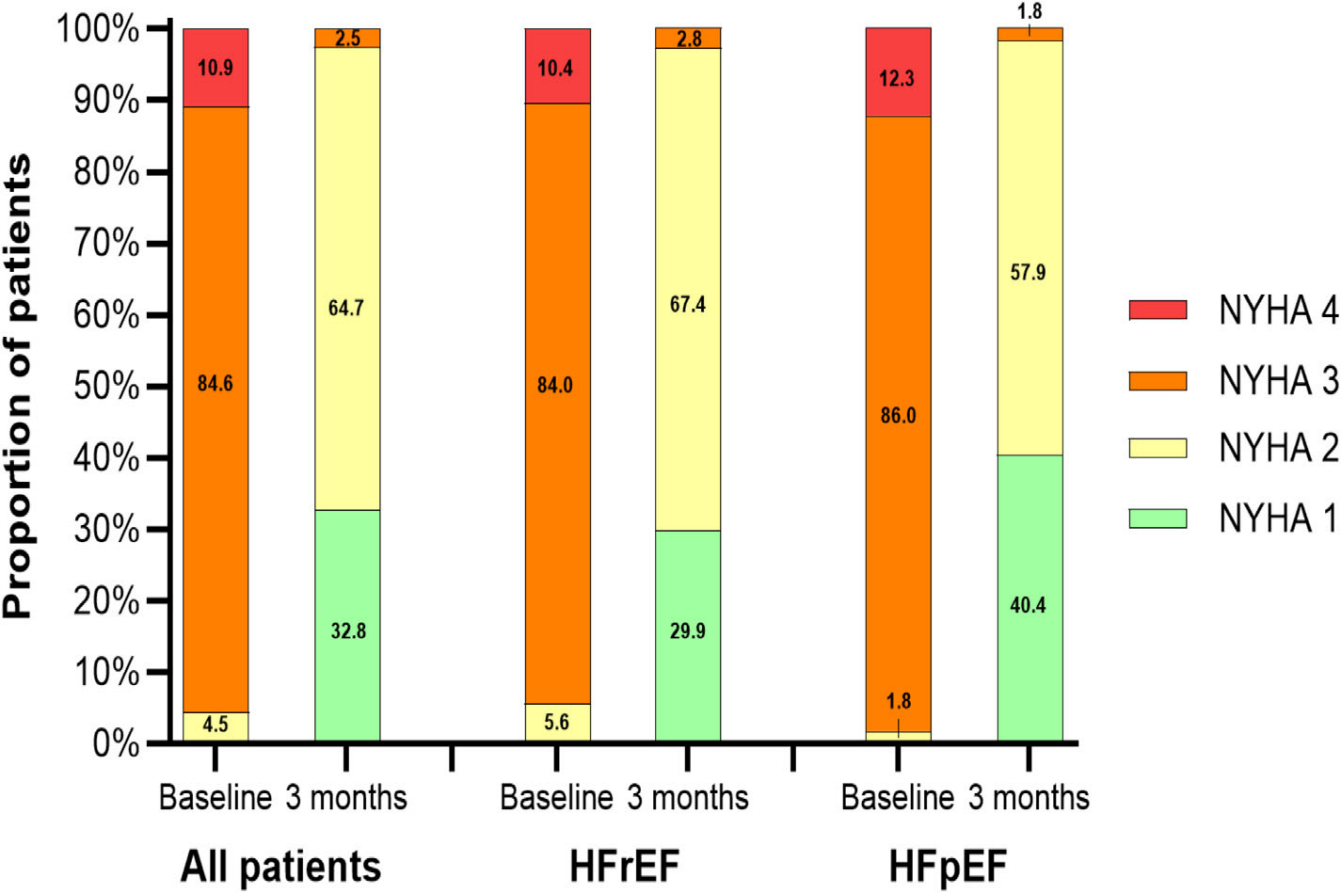
New York Heart Association (NYHA) classification before and 3 months after Carillon implantation. All patients (left panel), heart failure with reduced ejection fraction (HFrEF) (middle panel), and heart failure with preserved ejection fraction (HFpEF) (right panel). All baseline and 3‐month comparisons have a *P*‐value <0.001.

All patients returned for clinical, echocardiographic and laboratory follow‐up at 3 months. There were significant improvements in NYHA functional class by at least one category in 94% of patients, and 98% achieved NYHA I or II. Improvements in symptoms were not different between HFrEF and HFpEF patients (*Figure* *1*).

**Figure 2:**
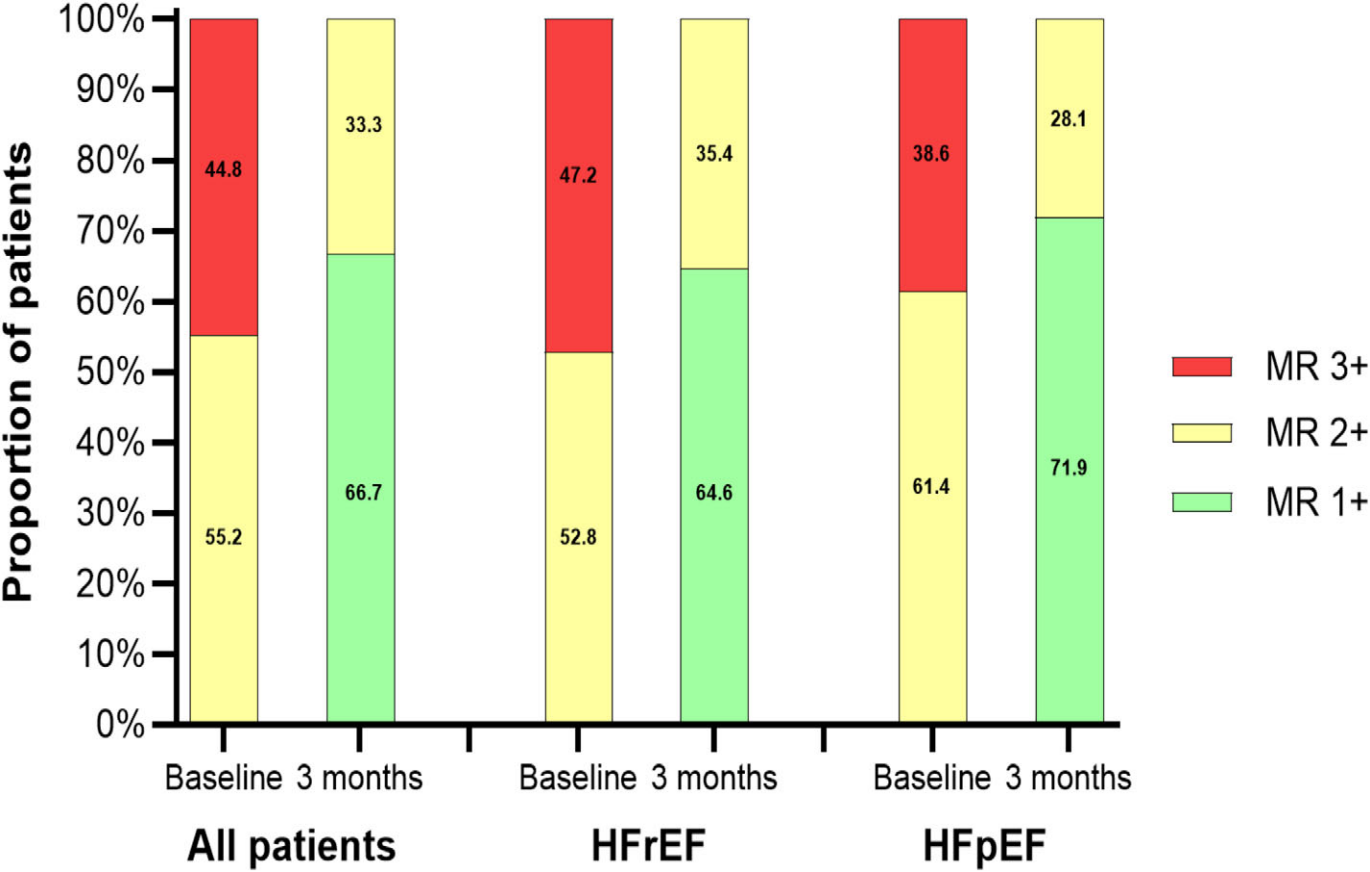
Mitral regurgitation (MR) grade before and 3 months after Carillon implantation. All patients (left panel), heart failure with reduced ejection fraction (HFrEF) (middle panel), and heart failure with preserved ejection fraction (HFpEF) (right panel). All baseline and 3‐month comparisons have a *P*‐value <0.001.

MR severity decreased by at least one grade in 91% of patients, with all classified as grade 1 + or 2 + at 3 months. In parallel, mean *vena contracta* width significantly decreased by 2.59 mm (95% CI: −2.72 to −2.45). There were no statistical differences in the frequency or degree of improvement between HFrEF and HFpEF (*Figure* *2*). Consistent with improvements in MR severity, left atrial dimensions demonstrated substantial reductions in both apical 2‐chamber (−7.4 cm^2^, 95% CI: −7.9 to −6.8) and 4‐chamber (−9.1 cm^2^, 95% CI: −9.6 to −8.5) views and systolic pulmonary artery pressure decreased by 9 mmHg (95% CI: −10 to −8) at 3 months.

There were statistically significant reductions in LVEDD in the overall cohort (−0.9 mm, 95% CI: −1.0 to −0.7). Diastolic function was also improved with the E/E′ ratio decreasing by 6.0 units (95% CI: −6.6 to −5.5) and the E‐wave velocity by 11 cm/s (95% CI: −13 to −9). Median BNP levels decreased from 2849 pg/mL (IQR: 1304–5838) at baseline to 1390 pg/mL (IQR: 683–3148) at 3 months, representing a decrease of 51% from baseline. There were no differences in the change in LV dimensions or other echocardiographic variables and BNP between the HFrEF and HFpEF groups (*Table* *2*).

**Table 2:** Echocardiographic and neurohormonal changes 3 months after Carillon device implantation

Endpoint	Mean change (95% CI)^a^			Mean difference (95% CI)^b^	*P*‐value^b^
	Total	HFrEF	HFpEF		
LVEDD (mm)	−0.9 (−1.0, −0.7)	−0.8 (−1.1, −0.6)	−0.9 (−1.3, −0.6)	0.3 (−0.1, 0.7)	0.18
Left atrial area (cm^2^)	−9.1 (−9.6, −8.5)	−9.2 (−9.9, −8.5)	−8.7 (−9.7, −7.6)	0.4 (−0.7, 1.5)	0.45
E/E′ ratio	−6.0 (−6.6, −5.5)	−6.2 (−6.8, −5.5)	−5.8 (−6.9, −4.7)	0.3 (−0.5, 1.2)	0.44
E‐wave (cm/s)	−11 (−13, −9)	−12 (−15, −10)	−9 (−14, −5)	−1 (−5, 3)	0.54
PAPsys (mmHg)	−9 (−10, −8)	−9 (−10, −8)	−9 (−11, −7)	1 (−1, 3)	0.27
Vena contracta (mm)	−2.59 (−2.72, −2.45)	−2.63 (−2.80, −2.46)	−2.47 (−2.69, −2.26)	−0.13 (−0.43, 0.17)	0.40
BNP (pg/mL)^c^	−1438 (−1900, −1059)	−1937 (−2553, −1431)	−659 (−959, −382)	111 (−230, 451)	0.52

Values are mean (95% CI) unless noted otherwise.

BNP, B‐type natriuretic peptide; E/E′ ratio, mitral inflow early velocity to mitral annular early diastolic velocity ratio; E‐wave, early mitral inflow velocity; HFpEF, heart failure with preserved ejection fraction; HFrEF, heart failure with reduced ejection fraction; LVEDD, left ventricular end‐diastolic diameter; PAPsy, systolic pulmonary artery pressure.

^a^ All change values over 3 months within each subgroup were statistically significant at *P* < 0.001.

^b^ Difference in change between HFpEF and HFrEF, values adjusted for baseline value.

^c^ Values are median (95% CI).

### Predictors of benefit and heterogeneity

**Figure 3:**
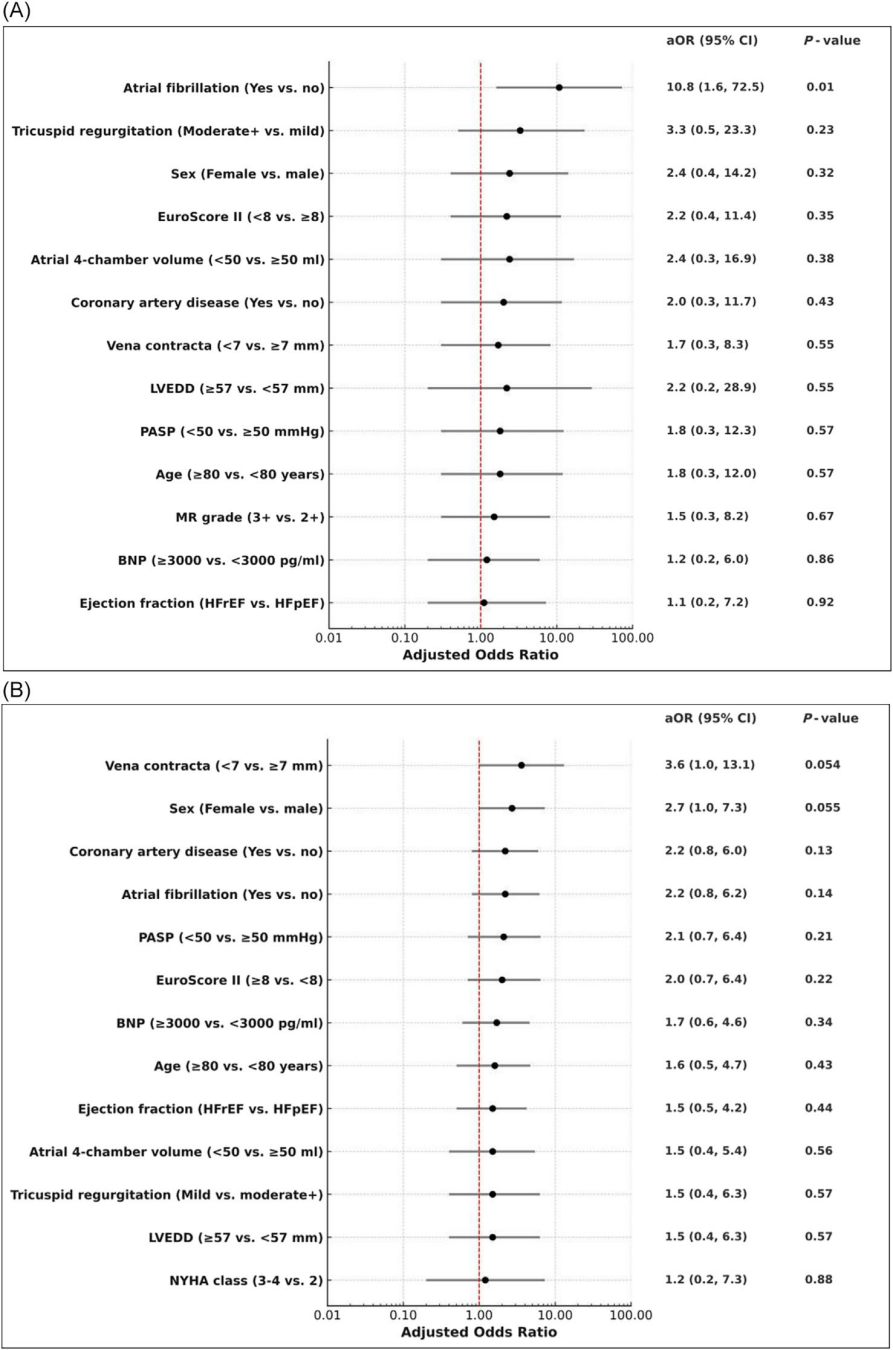
(A) Logistic regression analysis of factors associated with improvement in NYHA class (≥1 class) 3 months after Carillon implantation. (B) Logistic regression analysis of factors associated with improvement in mitral regurgitation grade (≥1 grade) 3 months after Carillon implantation.

After adjusting for baseline NYHA class, the presence of atrial fibrillation (adjusted odds ratio = 10.8; 95% CI 1.6, 72.5; *P* = 0.01) was independently associated with an improvement in NYHA class over 3 months (*Figure* *3* *A*). Comparing patients with atrial fibrillation with those without atrial fibrillation, the rate of symptomatic improvement based on NYHA class after CMCS was 97.5% vs. 80.5%. No other baseline variables showed a statistically significant association. Moreover, MR grade improvement was consistent across patient characteristics with no baseline variable significantly associated after adjusting for baseline MR grade (*Figure* *3* *B*). Notably, there was little heterogeneity in symptomatic improvement, change in MR severity by HF phenotype, or the need for circumflex intervention due to arterial compression.

## Discussion

The present analysis represents the largest single‐centre experience worldwide of consecutive, unselected patients undergoing CMCS implantation. Taken as a whole, these data demonstrate the short‐term safety and utility of the device but also reveal several important findings regarding short‐term efficacy. Firstly, the Carillon device demonstrated favourable 3‐month safety and effectiveness in both HFrEF and HFpEF populations with no difference in relevant outcomes between the two phenotypes of HF. Secondly, the device seems to function, at least in the short term, effectively in cohorts classically associated with worse outcomes and worse response to therapy, namely those with atrial fibrillation, more LV dilatation and those with more severe atrial dilatation. Thirdly, the present analysis demonstrated that the rate of serious complications is stable across the learning period. Finally, we have demonstrated that a circumflex artery compression can be treated by percutaneous coronary intervention with stent placement with no early adverse effect on symptomatic or mechanistic outcomes.

### Safety of CMCS in a wide range of patients

For patients and physicians a key criterion underpinning the use of a device to reduce MR in an older population with co‐morbidities is the safety of the procedure. The CMCS has reported safety in previous studies with a complication rate associated with device *placement* in the REDUCE‐FMR trial similar to that of placebo.^12^ Other studies have confirmed a high level of safety of both the procedure to cannulate the coronary sinus and place the device.^13^, ^14^, ^15^ However, these studies were carried out in the context of clinical trials in selected centres by experienced operators. The present data are the first describing the risks of the procedure in unselected, consecutive patients with eligibility for implantation determined entirely by their physicians in a standard of care pathway. Coronary sinus trauma was observed in fewer than 2% and did not preclude device implantation in any. Moreover, the major challenge associated with mitral annular reduction using an external approach, coronary artery compromise (with a > 50% narrowing), which occurred in around 10%, can be treated with circumflex intervention with no adverse impact on short‐term outcomes. Reassuringly, the complication rate was stable throughout the period which included any learning curve, testifying to the safety of the procedure.

### Clinical effectiveness

The effectiveness of the CMCS device on symptoms has been documented previously in the context of randomized and sham‐controlled trials.^13^, ^14^, ^15^ In the present, real‐world analysis, Carillon implantation was associated with improvements in NYHA class at 3 months. Moreover, there was little heterogeneity of effect on symptoms across a range of baseline variables. In fact there were greater symptomatic benefits seen in those with AF, a characteristic associated with lesser symptomatic and prognostic benefit in people receiving other device therapies for HFrEF.^22^, ^23^ If these data are supported in the ongoing randomized trials, patients with HF and AF might have an additional therapeutic option that works at least as well if not better than in those with sinus rhythm (SR).

### Mechanistic (echocardiographic and neurohormonal) endpoints

There were improvements in relevant mechanistic endpoints including clinically important reductions in left atrial size, diastolic variables and pulmonary artery pressure which were not appreciably different between HFpEF and HFrEF. These changes imply that treatment of MR using the CMCS is contributing to a normalization of the adverse haemodynamics in both HF phenotypes. Adverse atrial remodelling and impaired LA function are associated with poor outcomes in heart failure,^24^ including the worsening of symptoms,^25^ and are related to LV diastolic function and pulmonary artery pressure (PAP).^26^ Raised PAP itself is also associated with worse symptoms and outcomes in people with HF.^27^ The impact of MR severity on prognosis is influenced by systolic PAP.^28^ On the other hand, a high resting systolic PAP (>50 mmHg) is a marker of adverse risk even in those in whom SMR has been treated,^29^ while an improvement in the tricuspid annular plane systolic excursion/pulmonary artery systolic pressure ratio is independently associated with an improvement in symptoms and outcome.^30^ In COAPT, a reduction of ≥5 mmHg following TEER was associated with an improvement in prognosis.^25^ Hence our finding that both PAP and MR are improved in parallel with unbiased and clinically relevant reductions in B‐type natriuretic peptides following CMCS implantation is hypothesis‐generating: that the pathophysiological processes driving disease progression in both phenotypes of HF are interrupted and reversed by the treatment and that mitral annuloplasty using the CMCS could lead to improvement in prognosis in addition to symptoms.

While we saw statistically significant reductions in LV volume, these were modest in comparison to previous data^14^, ^15^, ^16^ possibly reflecting either the early nature of the assessment or the population being treated.

### Limitations

Several limitations of this study warrant consideration. First, as a single‐centre, observational study without a control group or core laboratory, we cannot exclude the possibility that the echocardiographic and symptom improvements may reflect regression to the mean, systematic bias or placebo effect. However, the BNP levels, done in a laboratory setting remote from the patients and therefore much less prone to bias, support the overall beneficial findings. Second, the follow‐up period was limited to 3 months and longer‐term follow‐up is required to establish the durability of the observed benefits. Third, we lacked a systematic assessment of quality of life measures or objective exercise capacity testing, which would have provided additional important patient‐centred outcomes. Fourth, the study population was predominantly elderly with a high prevalence of atrial fibrillation, potentially limiting generalizability to younger patients or those with different co‐morbidity profiles. Finally, we did not have baseline medications and were therefore not able to systematically evaluate changes in medication during follow‐up, which may have influenced outcomes independently of the device effect.

## Conclusions

In this single‐centre observational cohort study, CMCS implantation success was high, and associated with low complication rates, improvements in symptoms and echocardiographic variables, and accompanied by clinically significant reductions in BNP levels that were not different between HF phenotypes. These results demonstrate that the CMCS has the potential to address an important therapeutic gap not only for SMR patients with HFrEF but also for those with HFpEF who have not yet been systematically included in many clinical trials of transcatheter therapies.

## Conflict of interest

H.P.‐B. reports speaker fees and education meeting financial support from Cardiac Dimensions. F.J., N.R., M.L. and A.A. report no conflict of interest. K.K.W. held an NIHR (UK) Clinician Scientist Award, has received speaker fees and honoraria from Medtronic, Cardiac Dimensions, Novartis, Abbott, BMS, Pfizer, Bayer and has received unconditional research grants from Medtronic including one to support a PhD programme at the University of Leeds. H.J.H.'s work was supported by grants from the Deutsche Forschungsgemeinschaft, Cardiac Dimensions and Abbott, including speaker fees.

## Supporting information


**Table S1.** Periprocedural data*.

**Figure S1.** Cumulative sum learning curve of complications across 201 consecutive cases. The curve represents the cumulative deviation from the expected complication rate of 10.4%. Upward slopes indicate periods with more complications than expected, while downward slopes indicate fewer. The horizontal line at zero denotes the expected rate. The analysis did not demonstrate a consistent learning curve.
