# Supplementary material for: Indirect mitral annuloplasty in patients with reduced or preserved ejection fraction: A real‐world, single‐centre experience
Source: ESC Heart Fail. 2025 Nov 19;12(6):4410–8. doi: 10.1002/ehf2.70016 (PMC12719853; doi:10.1002/ehf2.70016)
Supplement: Supplementary file 1 — Table S1. Periprocedural data*. [file EHF2-12-4410-s002.doc]

**Supplementary Table 1: Periprocedural data***

| **Variable** | **Total**  **(n=201)** | **HFrEF**  **(n=144)** | **HFpEF**  **(n=57)** | **p-value** |
| --- | --- | --- | --- | --- |
| Procedure time (min) | 63 (51, 113) | 63 (51, 116) | 63 (52, 110) | 0.79 |
| Fluoroscopy time (min) | 16 (12, 24) | 16 (12, 24) | 16 (12, 28) | 0.82 |
| Contrast volume (ml) | 95 (64, 131) | 90 (61, 123) | 100 (78, 147) | 0.08 |
| Dose-area product (Gy⋅cm^2^) | 68 (47, 105) | 77 (47, 118) | 58 (40, 78) | 0.02 |
| Intervention-related mortality | 0% (0/201) | 0% (0/144) | 0% (0/57) | >0.99 |
| Complications (%)[n] | 10.4% (21/201) | 9.7% (14/144) | 12.3% (7/57) | 0.61 |
| PCI after circumflex artery compression | 9.5% (19/201) | 9.0% (13/144) | 10.5% (6/57) |  |
| Pericardial effusion after CS injury | 1.5% (3/201) | 0.7% (1/144) | 3.5% (2/57) |  |
| Hospital stay (days) | 3 (3, 4) | 4 (3, 4) | 3 (3, 4) | 0.47 |

*Values are median (interquartile range), or percentage (n/N).

PCI; percutaneous coronary intervention, CS; coronary sinus, HFpEF; heart failure with preserved ejection fraction, HFrEF; heart failure with reduced ejection fraction.
