# Supplementary material for: Indirect mitral annuloplasty in patients with reduced or preserved ejection fraction: A real‐world, single‐centre experience
Source: ESC Heart Fail. 2025 Nov 19;12(6):4410–8. doi: 10.1002/ehf2.70016 (PMC12719853; doi:10.1002/ehf2.70016)

**Supplementary Figure 1:** **Cumulative sum learning curve of complications across 201 consecutive cases.**

The curve represents the cumulative deviation from the expected complication rate of 10.4%.  Upward slopes indicate periods with more complications than expected, while downward slopes indicate fewer.  The horizontal line at zero denotes the expected rate.  The analysis did not demonstrate a consistent learning curve.


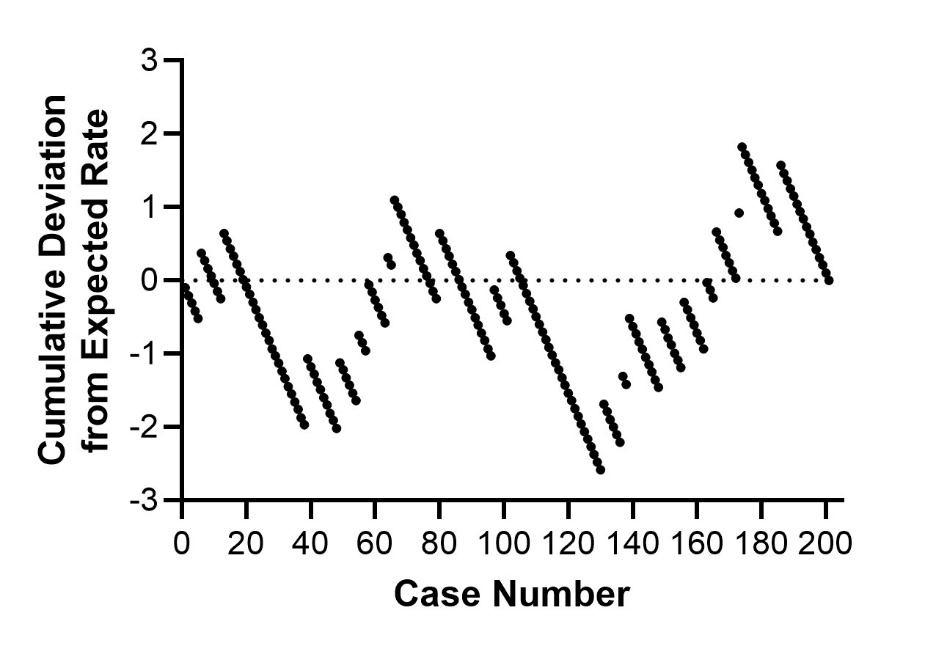

Supplement: Supplementary file 2 — Figure S1. Cumulative sum learning curve of complications across 201 consecutive cases. The curve represents the cumulative deviation from the expected complication rate of 10.4%. Upward slopes indicate periods with more complications than expected, while downward slopes indicate fewer. The horizontal line at zero denotes the expected rate. The analysis did not demonstrate a consistent learning curve. [file EHF2-12-4410-s001.docx]
